# Autophagic flux blockage in alveolar epithelial cells is essential in silica nanoparticle-induced pulmonary fibrosis

**DOI:** 10.1038/s41419-019-1340-8

**Published:** 2019-02-12

**Authors:** Xinyuan Zhao, Saisai Wei, Zhijian Li, Chen Lin, Zhenfeng Zhu, Desen Sun, Rongpan Bai, Jun Qian, Xiangwei Gao, Guangdi Chen, Zhengping Xu

**Affiliations:** 1 Institute of Environmental Medicine, Zhejiang University School of Medicine, Hangzhou, 310058 China; 2 Department of Occupational Medicine and Environmental Toxicology, School of Public Health, Nantong Unversity, Nantong, 226019 China; 3 The First Affiliated Hospital, Zhejiang University School of Medicine, Hangzhou, 310058 China; 4 State Key Laboratory of Modern Optical Instrumentation, Centre for Optical and Electromagnetic Research, JORCEP (Sino-Swedish Joint Research Center of Photonics), Zhejiang University, Hangzhou, 310058 China; 5 Collaborative Innovation Center for Diagnosis and Treatment of Infectious Diseases, Zhejiang University, Hangzhou, 310058 China

## Abstract

Silica nanoparticles (SiNPs) have been reported to induce pulmonary fibrosis (PF) with an unknown mechanism. Recently, the activation of autophagy, a lysosome-dependent cell degradation pathway, by SiNPs has been identified in alveolar epithelial cells (AECs). However, the underlying mechanism and the relevance of SiNPs-induced autophagy to the development of PF remain elusive. Here, we report that autophagy dysfunction and subsequent apoptosis in AECs are involved in SiNPs-induced PF. SiNPs engulfed by AECs enhance autophagosome accumulation and apoptosis both in vivo and in vitro. Mechanically, SiNPs block autophagy flux through impairing lysosomal degradation via acidification inhibition. Lysosomal reacidification by cyclic-3′,5′-adenosine monophosphate (cAMP) significantly enhances autophagic degradation and attenuate apoptosis. Importantly, enhancement of autophagic degradation by rapamycin protects AECs from apoptosis and attenuates SiNPs-induced PF in the mouse model. Altogether, our data demonstrate a repressive effect of SiNPs on lysosomal acidification, contributing to the decreased autophagic degradation in AECs, thus leading to apoptosis and subsequent PF. These findings may provide an improved understanding of SiNPs-induced PF and molecular targets to antagonize it.

## Introduction

Nanoparticles (NPs) defined as particles having at least one dimension below 100 nm have been applied widely in the last decade in industry and medicine^1^. Among those NPs, silica nanoparticles (SiNPs) are one of the most widely used and closely related to our daily life containing drug delivery, cosmetics and paint, etc^2–4^. The increasing use of NPs has raised concerns about their human and environmental risks. Because their physicochemical properties are different from large particles, NPs may potentially result in toxic effects with yet unknown mechamisms. The respiratory system is considered to be one of the main routes by which NPs access human body^5^. Inhalation of these ambient ultrafine particles can result in pulmonary oxidative stress, inflammation, and ultimately cell death^1^. Despite intense investigations, current knowledge of physiological effects of SiNPs on biological barriers and the underlying molecular mechanisms remains fragmented.

Pulmonary fibrosis (PF) is the ultimate result of a large and heterogeneous group of lung disorders known as interstitial lung diseases. It is characterized by excessive accumulation of extracellular matrix, leading to a decline in lung function^6^. Many nano-size materials, including nanoparticulate titanium dioxide, multi- or single-walled carbon nanotubes, as well as SiNPs, have been found to cause PF^7–11^. The dysregulation of fibroblasts activities including migration, proliferation, secretion, and myofibroblast differentiation is central to the development of PF. Some NPs, including SiNPs, could activate macrophages to induce inflamatory cytokines secretion^7–9^. These cytokines could triger uncontrolled activation of fibroblasts, which untimately induces PF development. Current paradigms point to alveolar epithelial cells (AECs) injury as another critical event during the pathogenesis of PF. Surrounding the injured AECs, fibroblasts and myofibroblasts form the fibroblastic foci and deposit large amounts of extracellular matrix, thereby destroying the normal alveolar architecture^12^. Although there are studies showing that AECs could uptake NPs in vivo and in vitro, no study has examined the role of AEC damage in NPs-induced PF^13,14^.

As a genetically programmed pathway for the turnover of cellular components, autophagy has emerged as a crucial process for cellular homeostasis. During autophagy, cytosolic substrate or “cargo” is sequestered into double-membrane vesicle (autophagosome), fusing with lysosome for internal materials degradation^15^. Accumulating evidences suggests that dysregulation of autophagy plays an important role in PF. The mammalian target of the rapamycin (mTOR) signaling pathway, a core signaling pathway to regulate autophagy, has been reported to participate in the process of PF. Using a transgenic mouse model, Gui et al. found that mTOR overactivation in AECs compromised autophagy in the lung and was involved in the pathogenesis of bleomycin-triggered PF^16^. Similarly, Singh et al. reported that deficient autophagy resulted in upregulation of TGF-β1, a key fibrotic driver in PF, promoting PF development^17^. Additionally, autophagy-deficient mice displayed a significantly greater inflammatory response after bleomycin treatment^18,19^. Collectively, these findings support that impaired autophagy may contribute to PF. However, the specific role and underlying mechanism of autophagy, especially in AECs, during NPs-induced PF are still undefined.

In this study, we investigated in detail the dysregulation of autophagy by SiNPs in AECs and defined its contribution to SiNPs-induced PF. Our findings provide the first evidence that SiNPs block autophagic flux in ACEs, contributing to subsequent PF.

## Materials and methods

### Synthesis of silica nanoparticles

The micelles was used to dissolve a certain number of sulfobernteinsaure-bis-2-ethylhexy ester natriumsalz (Aerosol-OT) and 1-butanol in total 10 mL of DI water under energetic vigorous magnetic stirring. Hundred microliter triethoxyvinylsilane triethoxyvinylsilan (VTES) was added to micellar system mentioned above after 30 min, and was stirred for another 1 h. Then, SiNPs were precipitated after addition of 10 μL of (3-aminopropyl) triethoxysilane (APTES) and stirred at room temperature for another 20 h. After successful formation of the SiNPs, excess Aerosol-OT, co-surfactant 1-butanol, VTES, and APTES were removed by dialyzing the solution against DI water in a 12–14 kDa cutoff cellulose membrane for 50 h. The dialyzed solution was then filtered by a 0.45 μm filter for further experiments.

### Charicterization of silica nanoparticles

Transmission electron microscope (TEM) was taken by a JEOL JEM-1200EX transmission electron microscope for nanoparticles. SiO_2_ NPs were dispersed in Roswell Park Memorial Institute (RPMI)-1640 medium (Invitrogen, Carlsbad, CA, USA) for 0 and 24 h and then subjected to dynamic light scattering (DLS) and zeta-potential measurements using the instrument Zetasizer Nano ZS-90 (Malvern Instruments, Orsay, France). X-ray diffraction (XRD) was performed using Bruker D8 Advance diffractometer (Bruker, Billerica, MA, USA) and using CuKα (*λ* = 1.54 Å) as a radiation source.

### Plasmids, reagents, and antibodies

The plasmids GFP-LC3, CFP-LC3, and YFP-LAMP1 were gifts from Dr. Wei Liu (Department of Biochemistry and Molecular Biology, Zhejiang University School of Medicine)^20,21^. The SiNPs were provided by Dr. Jun Qian (State Key Laboratory of Modern Optical Instrumentation, Zhejiang University) and suspended in phosphate-buffered saline (PBS)^22^. Before use, the SiNPs solution was filtered through 0.22 μm cutoff membrane filter, then its concentration was detected. To mark SiNPs, Nile Red was added in it^23^. The chemicals rapamycin (R8781), chloroquine (CQ) (C6628), 3-methyladenine (3-MA) (M9281), cycloheximide (C4859), and EGF (E9644) were from Sigma. TRIzol reagent (3220621) and DNA Transfection Reagent (2102-100) were from SuperFectin. Bodipy-FL-pepstatin A (P12271) was from Invitrogen. DND Green-189 (L7535) was purchased from Molecular Probes. The blocking serum and Alexa Fluor 488-conjugated goat anti-rabbit second antibody were from Zhongshan Golden Bridge Biotechnology. 8-CPT-cAMP (sc-201569) and IBMX (sc-201188) were from Santa Cruz. The forskolin (S1612) was purchased from Beytime.

The following primary antibodies were used: anti-LC3 (Sigma, L7543), anti-p62 (Sigma, P0067), anti-ACTB (Sigma, A5316), anti-cleaved-Caspase3 (Cell Signaling, 9664), anti-EEA1 (Cell Signaling, 3288), anti-EGFR (Cell Signaling, 4267), anti-α-SMA (Proteintech, 14395-1-AP), anti-Bcl-2 (Proteintech, 12789-1-AP), anti-Bax (Proteintech, 23931-1-AP), and anti-CTSD (BD Biosciences, 610800).

### Animal treatment

All animal experiments were performed according to the ethical guidelines by the Ethics Committee of Laboratory Animal Care and Welfare, Zhejiang University School of Medicine. To generate pulmonary fibrosis animal model, the Institute of Cancer Research (ICR) male mice at 6 weeks were anesthetized with chloral hydrate, instilled with SiNPs at the dose of 5.0 mg/kg, a dose that has been reported to induce fibrogenic effect^24^, and sacrificed at indicated time. To determine the role of autophagic flux in SiNPs-induced pulmonary fibrosis, mice were given with or without rapamycin (10 mg/kg/day after SiNPs instillation). Biopsies of lung tissues were fixed with 10% paraformaldehyde in sterile phosphate-buffered saline, embedded in paraffin wax, and sectioned at 5 μm for further staining. Total protein and RNA from tissues were isolated, and stored at −80 °C for determination.

### Lung compliance

Lung compliance was measured by Buxco system (Buxco, RC system). Briefly, mice were anesthetized and placed in a whole body plethysmograph connected to a Harvard rodent ventilator. Data were collected and analyzed using the Biosystems XA software package.

### Lung/body coefficient

Mice were weighted (body weight) before being sacrificed. Their lungs were harvested and weighted (lung wet weight), then the lung/body coefficient was calculated as lung/body coefficient = lung wet weight [g]/body weight [g] × 100%.

### HYP level analysis

The collagen content in the lung tissue was determined by analysis of the HYP level which was measured by spectrophotometer at 550 nm with detection kit (Nanjing Jiancheng Bioengineering Institute, A030-2).

### Quantitative real-time polymerase chain reaction (qRT-PCR) analysis

Total RNA was isolated with TRIzol reagent following the manufacturer’s protocol. The qRT-PCR analysis was performed as described before^25^. Briefly, real-time quantitative PCR analysis was performed in 10-μL reactions using SYBR GREEN PCR Master Mix (Applied Biosystems). The related mRNA level was normalized to the *β-actin* mRNA level. The specific primer sequences were listed in Table 1.

**Table 1:** DNA sequences of primers for polymerase chain reaction

Gene name	Primer name	Sequence
Human *β-actin*	H-ACTB-F	5′-CACGATGGAGGGGCCGGACTCATC-3′
	H-ACTB-R	5′-TAAAGACCTCTATGCCAACACAGT-3′
Human *LC3b*	H-LC3b-F	5′-TTGGTGAACGGACACAGCAT-3′
	H-LC3b-R	5′-CGTCTCCTGGGAGGCATAGA-3′
Mouse *β-actin*	M-ACTB-F	5′-GCAGATGTGGATCAGCAAGC-3′
	M-ACTB-R	5′-AGCTCAGTAACAGTCCGCC-3′
Mouse *CTGF*	M-CTGF-F	5′-AGACCTGTGCCTGCCATTAC-3′
	M-CTGF-R	5′-ACGCCATGTCTCCGTACATC-3′

### Transmission electron microscopy

After treatment as indicated, lung tissues or A549 cells were fixed in 2.5% glutaraldehyde at 4 °C overnight. After dehydration, ultrathin section was embedded and stained with uranyl acetate/lead citrate. Image was captured under a transmission electron microscopy (FEI Company, TECNAI 10).

### Masson, Sirius red, TUNEL assay, and immunohistochemistry staining

The tissue slice with 5 μm thickness was subjected to Masson staining or Sirius red staining for identification of collagen fibers^26^. Apoptosis cells in the tissue section were detected by TUNEL using IHC assay with a commercial apoptosis kit (11684817910, Roche), according to the supplier’s instruction. For IHC staining, the tissue section was deparaffinized and rehydrated, and boiled in 10 mM citrate buffer for 30 min for antigen retrieval. Endogenous peroxidase was blocked with 3% hydrogen dioxide for 10 min. The slide was blocked in 3% bovine serum albumin (BSA) at room temperature for 1 h, incubated with corresponding primary antibody (LC3, 1:1000; p62, 1:200; or α-SMA, 1:200) at 4 °C overnight, then incubated with anti-rabbit secondary antibody for 1 h, and visualized with 3,3-diaminobenzidine substrate (Zhongshan Golden Bridge Biotechnology, #PV-9000-D). Finally, the section was counterstained with hematoxylin.

### Cell culture, transfection, and autophagy induction

The human lung epithelial cells (A549) were kindly provided by Dr. Yuehai Ke (Department of Basic Medical Sciences, Zhejiang University School of Medicine), and were grown as a monolayer in Royal Park Memorial Institute (RPMI) 1640 (Invitrogen, C22400500BT) supplemented with 10% fetal bovine serum (FBS, Gibco, 1027-106) at 37 °C in an humidified atmosphere of 95% air and 5% CO_2_. Transient transfection was performed using DNA Transfection Reagent (Invitrogen, 11668-019) according to manufacturer’s instruction.

For autophagy induction, the cells were incubated in starvation medium (1% BSA, 140 mM NaCl, 1 mM CaCl_2_, 1 mM MgCl_2_, 5 mM glucose, and 20 mM HEPES, pH 7.4) at 37 °C for 2 h, or cultured in 10% FBS of RPMI 1640 with 200 nM rapamycin for 24 h.

### Immunoblotting analysis

Total proteins were extracted from cells or lung tissue after lysed with lysis buffer, quantified by bicinchonininc acid (BCA) protein assay kit (Beyotime, P0009), and applied to immunoblotting analysis as described previously^27^. The equal amounts of total proteins were separated by sodium dodecyl sulfate polyacrylamide gel electrophoresis and transferred to nitrocellulose membrane. The membrane was blocked with 3% BSA in TBST buffer for 2 h. Next, the blot was incubated with corresponding primary antibody (anti-LC3, 1:1000; anti-p62, 1:1000; anti-EGFR, 1:1000; anti-ACTB, 1:2000; anti-GAPDH, 1:2000; or anti-CTSD, 1:500) at 4 °C overnight followed by incubation with IR-Dye conjugated secondary antibody (Goat anti-Mouse, Li-COR Biosciences, B80908-02, 1:10,000; or Goat anti-Rabbit, Li-COR Biosciences, B81009-02, 1:10,000) for 1 h. Finally, the specific bands were analyzed using an Odyssey infrared imaging system (Li-COR Biosciences) and then quantified using densitometry.

### Immunofluorescence staining

The cells cultured on coverslips were washed three time in 1 × PBS, fixed with 4% paraformaldehyde for 15 min at 4 °C, and permeabilized with 0.2% Triton X-100 for 15 min at 4 °C. The non-specific binding was blocked by goat serum (Zhongshan Golden Bridge Biotechnology, #ZLI-9021) at room temperature for 2 h. Then the cells were incubated with primary antibodies at 4 °C overnight (anti-LC3, 1:200; anti-p62, 1:200; anti-EEA1, 1:100; anti-EGFR, 1:1000), and incubated with Alexa Fluor 488-conjugated goat anti-rabbit second antibody (1:1000) for 1 h. The sample was further incubated with 4′,6-diamidino-2-phenylindole (DAPI, Sigma, D9542) for 15 min to stain nuclei. Finally, the cover slip was mounted onto a glass slide with 87% glycerol, and the immunofluorescence staining was visualized with a confocal microscope (Nikon, A1R).

### EGFR degradation assay

The cells treated with SiNPs at concentration of 50 μg/mL for 24 h were incubated in serum-free DMEM with 10 μM cycloheximide for another 4 h. Then, the cells were treated with 100 ng/mL EGF for different durations. The EGFR expression level was evaluated by cellular immunofluorescence assay, or immunoblotting assay.

### Determination of lysosomal acidification

For lysosomal acidification assay, the cells were incubated with the 1.0 μM DND Green-189 (Invitrogen, L7525) for 30 min at 37 °C. After staining, cells were washed with PBS twice, and cellular fluorescence was evaluated by flow cytometry (Beckman Coulter, FC500MCL) or imaged by a fluorescent microscope (Nikon, ECLIPSE Ti) to quantify lysosomal acidification.

### Bodipy-FL-pepstatin A staining

For mature CTSD staining, the cells were washed with PBS for twice, and were incubated with 1 mM Bodipy-FL-pepstatin A for 1 h at 37 °C. After washing with PBS, cells were imaged by a confocal microscope (OLYMPUS, BX61W1-FV1000) to quantify relative pepstatin A intensity.

#### Lysosomal reacidification

To reacidify lysosome, a cAMP cocktail was used. It consisted of 8-CPT-cAMP (500 μM), IBMX (100 μM), and forskolin (10 μM).

### Cell viability analysis

The effects of various treatments on cell viability were determined by Cell Counting Kit-8 (CCK-8) reagent (DOJINDO, CK04). Briefly, 10 μL of CCK8 reagent was added to each well of cells plated in 96-well plate and incubated for another 2 h at 37 °C. The optical density (OD) at 450 nm was measured by using a Microplate Reader (Thermo Scientific, VaripsKan Flash).

### Apoptosis assay

Apoptosis analysis was performed by Annexin V staining. Briefly, cells were resuspended and incubated with Annexin V (MultiSciences Biotech Co., Ltd, 5300117) for 5 min in the dark. The sample was analyzed by flow cytometry (Beckman Coulter, FC500MCL), and the percentage of Annexin V positive cells was counted.

### Statistical analysis

The SiNPs size was measured by Image J software. The fluorescence intensities and protein colocalization coefficients were quantified by the MetaMorph software. The 3D structure and size measurement of lysosome were carried out with Imaris software. Comparison between two groups (Control and SiNPs) for statistical difference were performed with Student’s two-tailed *t* test. One-way ANOVA was used to determine statical significance for multiple groups analysis. For lysosome volume, Wilcoxon rank sum test was used. All statistical analyses were performed using GraphPad Prim version 6.0. A *P* value ≤ 0.05 was considered significant. Differences were considered statistically significant if *P* < 0.05 (*), *P* < 0.01 (**), or not significant (NS).

## Results

### SiNPs induce PF in mice

**Fig. 1:**
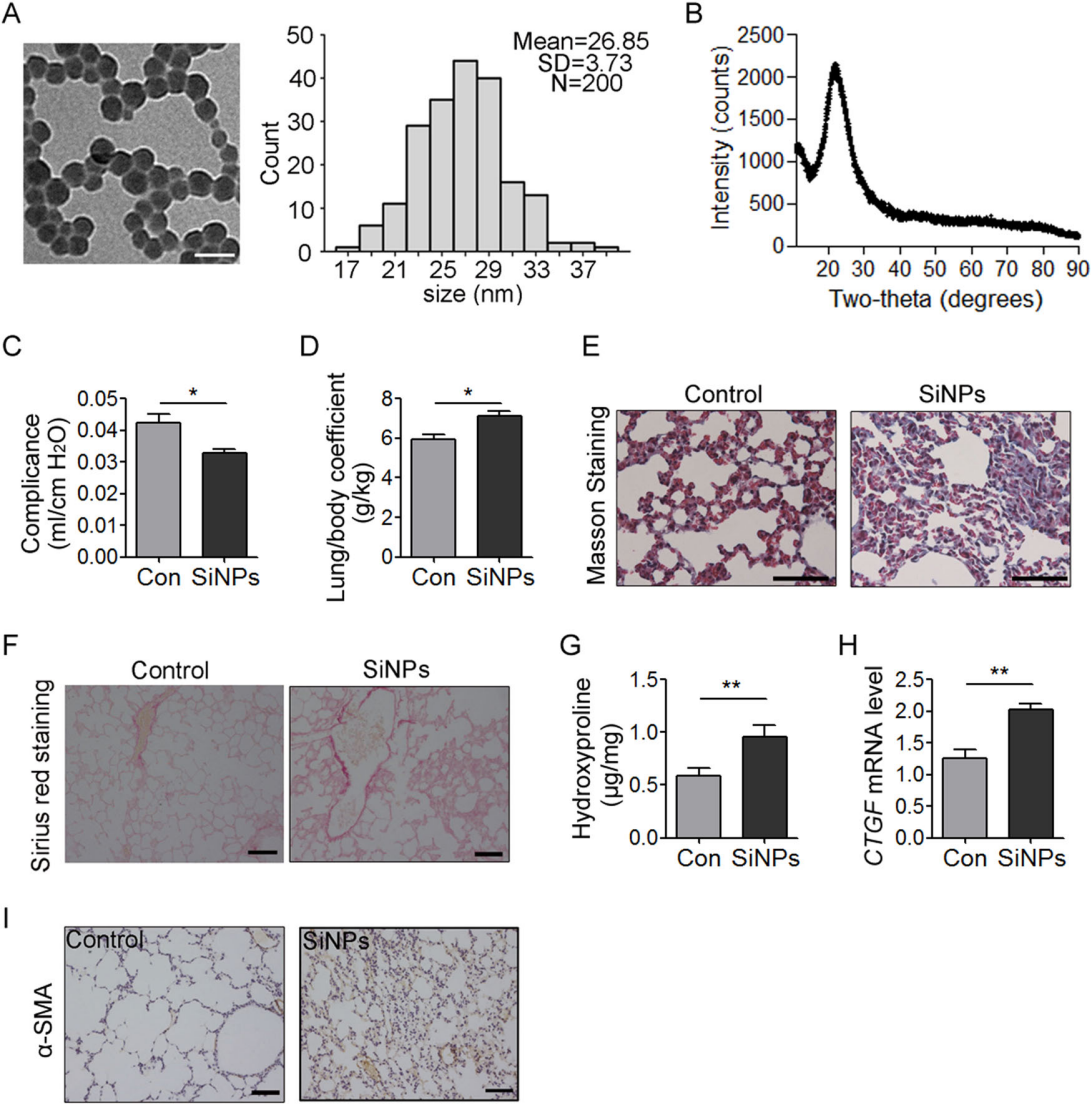
SiNPs induce pulmonary fibrosis (PF). **a** SiNPs were imaged by TEM. SiNPs size showed approximately normal distribution, and the average SiNPs size was about 26.85 nm. Scale bar: 50 nm. **b** XRD analysis of SiNPs using CuKα (*λ* = 1.54 Å) as a radiation source. **c**–**i** The mice were instilled once with SiNPs and sacrificed 1 month later. **c** Lung compliance was determined by Buxco system. **d** The weights of lung and body were recorded to determine the lung/body coefficient. **e**, **f** The lung tissue sections were stained by Masson staining (**e**) or Sirius red staining (**f**) to identify collagen fibers. **g** The hydroxyproline (HYP) content in lung tissue was assessed by spectrophotometry. **h** The relative *CTGF* mRNA level was measured by qRT-PCR. **i** The expression level of α-SMA was detected by immunohistochemistry (IHC). Data are presented as mean ± SEM (*n* = 5 for each group). *P < 0.05, **P < 0.01. Scale bars: 100 μm

We prepared silica SiNPs as described in methods with an average diameter about 26.85 nm (Fig. 1a). The DLS of SiNPs in culture media RPMI-1640 were 37.53 ± 1.98 nm, and exhibited good stability in media since the size did not increase after 24 h of incubation (38.12 ± 2.37 nm, Table 2). In addition, the results of zeta potentials of SiNPs in our study were −30.40 ± 0.83 and −28.75 ± 0.59 mV, respectively. The X-ray diffraction pattern of the silica nanoparticles indicates a peak at 2*θ* = 22°, which reveals the amorphous nature of the silica nanoparticles. Further, the XRD pattern confirms the absence of any ordered crystalline structure (Fig. 1b).

**Table 2:** DLS, zeta potential, and polydispersity index of SiNPs in RPMI 1640 at different time points

Time (h)	DLS (nm)	Zeta potential (mV)	Polydispersity index
0	37.53 ± 1.98	−30.40 ± 0.83	0.295 ± 0.03
24	38.12 ± 2.37	−28.75 ± 0.59	0.301 ± 0.04

To determine whether these SiNPs could induce PF, mice were instilled with SiNPs at 5 mg/kg. The mice were sacrificed at one month after instillation, and lung compliance and lung/body coefficient, as important indexes of lung function, were evaluated. The results showed that comparing to the control group, pulmonary compliance was significantly reduced (Fig. 1c), and lung/body coefficient was significantly increased in SiNPs-treated group (Fig. 1d), indicating an impairment of lung function. Furthermore, Masson’s staining and Sirius red staining revealed an increased collagen deposition in SiNPs-exposed mice lung tissue (Fig. 1e, f). SiNPs treatment significantly increased the expression of hydroxyproline (HYP), a major constituent of collagen, and connective tissue growth factor (CTGF), which could induce collagen synthesis, in mice lungs (Fig. 1g, h), further demonstrating collagen accumulation in lung tissues^28^. Finally, α-smooth muscle actin (α-SMA) expression level, as active fibrotic indicator, was significantly upregulated in the lung tissues of SiNPs-treated mice (Fig. 1i)^29^. Taken together, these data indicate that SiNPs do induce PF in mice under current experimental conditions.

### SiNPs enter AECs via endosome/lysosome pathway

**Fig. 2:**
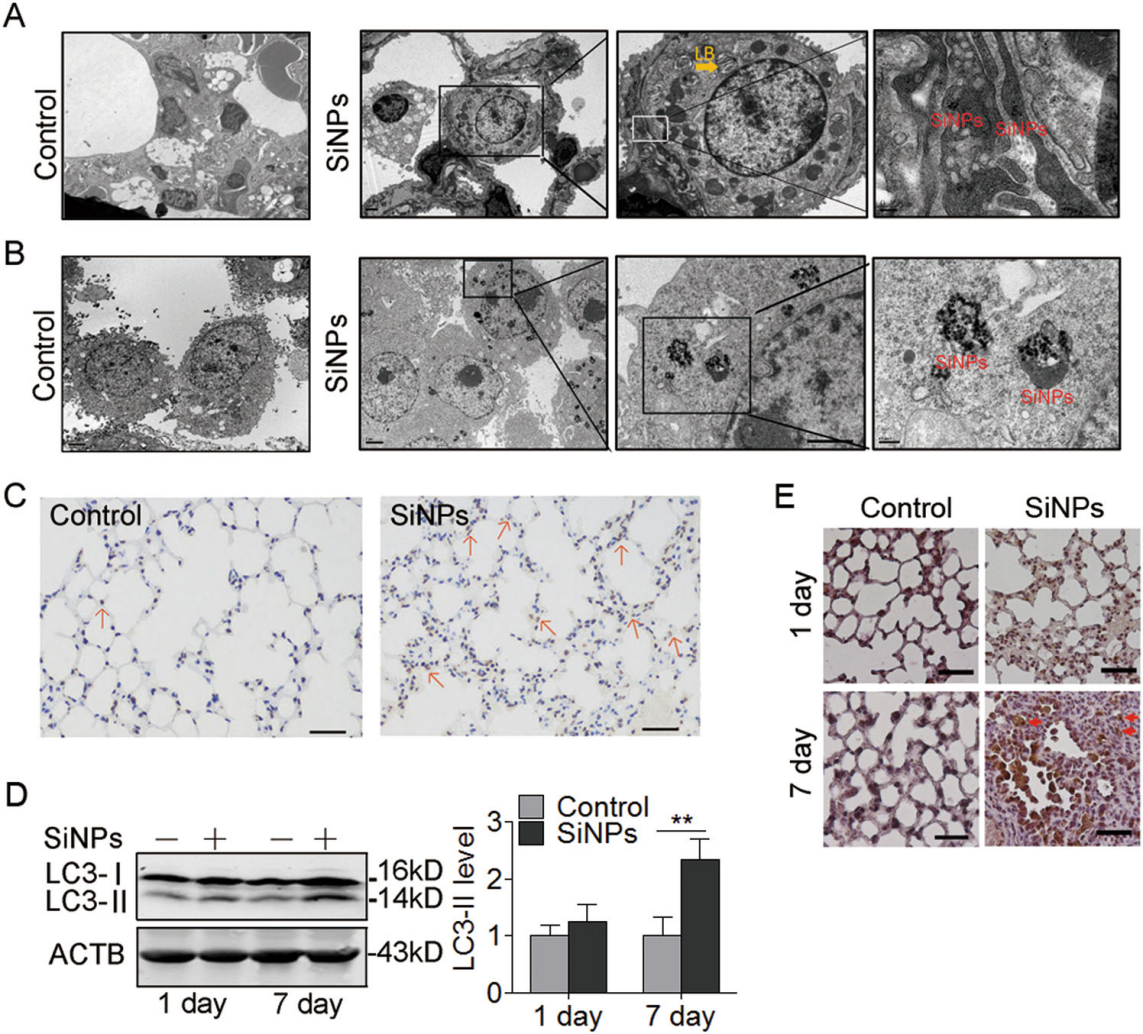
SiNPs induce apoptosis and autophagosome accumulation in mouse lung tissue. **a** The mice were instilled once with SiNPs (100 μg) and harvested 1 day later. The lung tissue was imaged by TEM. LB, lamellar body. Scale bars: 200 nm. **b** The cellular location of SiNPs in A549 was imaged by TEM. Scale bars: 200 nm. **c** Cell apoptosis for lung tissue from mice with or without SiNPs treatment was detected by TUNEL assay 1 month after instillation. Red arrows: TUNEL-positive cells. Scale bars: 100 μm. **d**, **e** The mice were instilled with PBS or SiNPs, and harvested at the indicated time. **d** LC3 protein expression of lung tissue was detected by western blot, and the right panel shows the statistics for the relative LC3-II protein level. **e** LC3 expression was detected by IHC at the indicated time after instillation. Scale bars: 100 μm. All the quantitative data are presented as mean ± SEM (*n* = 5 for each group), **P < 0.01

To test the hypothesis that the AECs damage contributes to SiNPs-induced PF, we first examined that AECs can endocytose SiNPs. The transmission electron microscopy (TEM) image showed that SiNPs were in type II AECs one day after instillation (Fig. 2a). To further confirm our observation, A549 cells were exposed to SiNPs at a concentration of 50 μg/mL for 24 h and imaged by TEM. As expected, A549 cells also engulfed SiNPs (Fig. 2b). Next, we monitored the subcellular localization of SiNPs at different time points. The results showed that SiNPs mainly colocalized with early endosome antigen 1 (EEA1), an early endosome marker, after 30 min treatment (Figure S1A, Supporting Information). With the treatment time extends, more and more SiNPs were colocalized with lysosomal-associated membrane protein 1 (LAMP1), a lysosome marker, accompanied by decreased colocalization with EEA1 (Figure S1B, Supporting Information), suggesting a translocation from early endosome to lysosome. These data indicated that SiNPs could penetrate AEC membrane and enter the lysosomes via endosome/lysosome pathway.

### SiNPs stimulate autophagosome accumulation and cell apoptosis both in vitro and in vivo

To reveal the biological consequence of cellular SiNPs, cell apoptosis was determined by terminal deoxynucleotidyl transferase-mediated nick end labeling (TUNEL) assay in vivo. The result showed obvious TUNEL-positive staining in SiNPs-treated group, indicating that SiNPs exposure induced cell apoptosis in lung tissue (Fig. 2c). Next, we detected the expression level of microtubule-associated protein 1 light chain 3 (MAP1LC3), an autophagosome marker, in lung tissues by Western blot, and found that LC3-II expression was increased in SiNPs-treated mice (Fig. 2d). Immunohistochemistry (IHC) staining confirmed that the LC3 protein accumulated in lung tissues from SiNPs-treated mice (Fig. 2e).

**Fig. 3:**
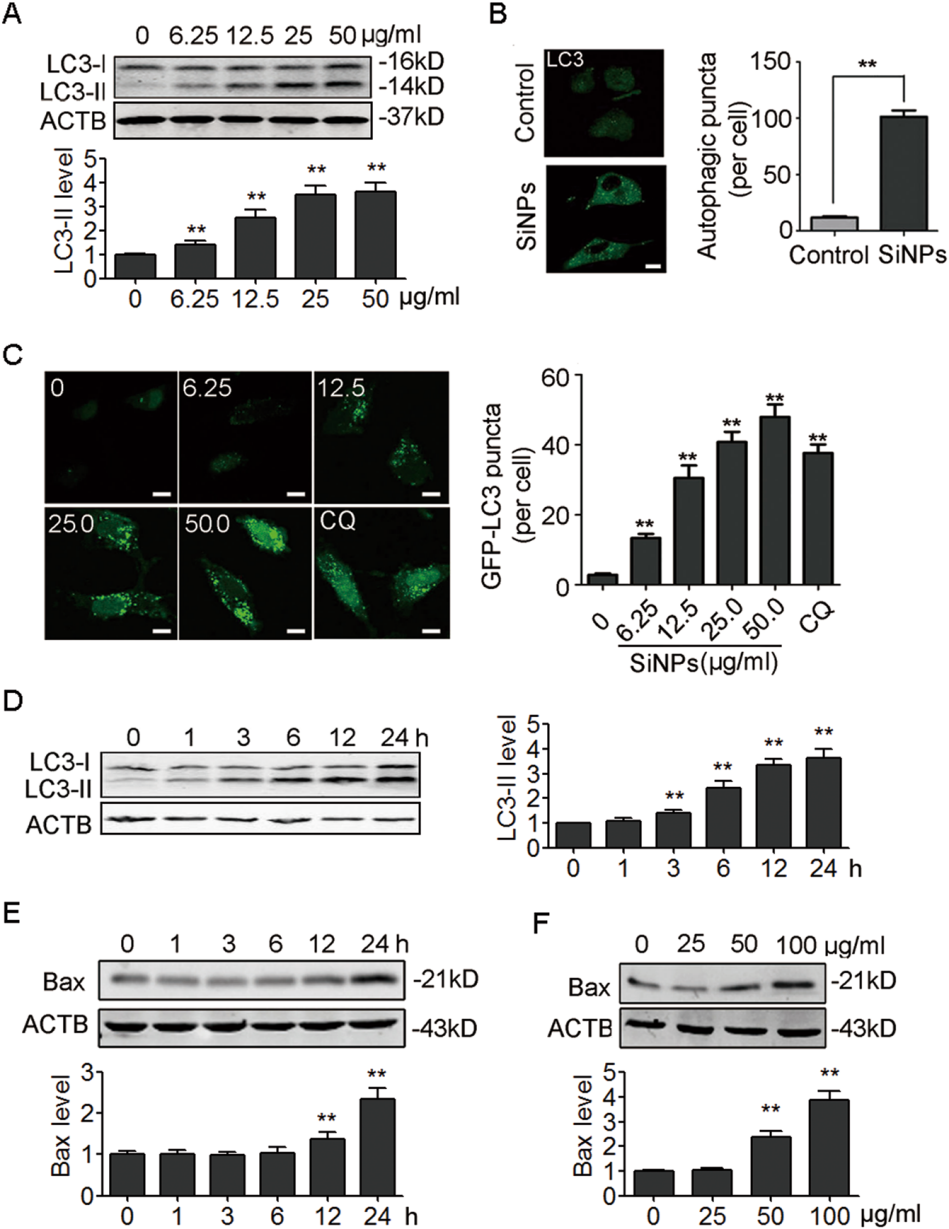
SiNPs stimulate autophagosome accumulation and apoptosis in A549 cells. **a** A549 cells were treated with different concentrations of SiNPs for 24 h, and the cellular LC3 expression levels were detected by western blot. The bottom panel shows the statistics for the relative LC3-II protein level. **b** A549 cells were treated with SiNPs at 50 μg/mL for 24 h, and the cellular LC3 expression level was evaluated by indirect immunofluorescence (IF) assay. Quantification shown on the right represents the average number of autophagic puncta per cell. *n* = 50. **c** A549 cells transiently expressing GFP-LC3 were exposed to different concentrations of SiNPs for 24 h, or treated with 20 μM chloroquine (CQ) for 6 h as a positive control, and were imaged by confocal microscopy. The graph on the right shows the statistics for the average number of GFP-LC3 puncta per cell. *n* = 50. **d** A549 cells were treated with SiNPs at 50 μg/mL for different time as indicated, and cellular LC3 expression was assessed by western blot. The right panel shows the statistics for the relative LC3-II protein level. **e** A549 cells were treated with SiNPs at 50 μg/mL for different time as indicated, and cellular Bax expression was assessed by western blot. The bottom panel shows the statistics for the relative Bax protein level. **f** A549 cells were treated with different concentration of SiNPs for 24 h, and cellular Bax expression was assessed by western blot. The bottom panel shows the statistics for the relative Bax protein level. **P < 0.01. Scale bars: 5 μm

We further performed in vitro studies using A549 cells. The results showed that SiNPs increased LC3-II expression in a dose-dependent manner in A549 cells (Fig. 3a). SiNPs-induced LC3-II expression was also observed in another lung epithelial cells BEAS-2B (Figure S2A). Consistently, immunofluorescence (IF) staining revealed more LC3-positive punctuated structures in SiNPs-treated cells (Fig. 3b). Similarly, increased green fluorescent protein (GFP) tagged LC3 (GFP-LC3) punctuated structures were found in GFP-LC3-expressing A549 cells after SiNPs treatment (Fig. 3c). SiNPs induced significant autophagosome accumulation within the first 3 h trearment (Fig. 3d). Higher concentration of SiNPs (50 µg/ml) also upregulated the espression of B-cell lymphoma 2 (Bcl-2)-associated X protein (Bax), an indicator of apoptosis (Fig. 3e). In addition, exposure to SiNPs led to apoptosis in a dose-dependent way as indicated by gradually increased Bax (Fig. 3f). Taken together, these data demonstrate that SiNPs induce autophagosome accumulation and apoptosis in AECs.

### SiNPs inhibit autophagosome degradation

**Fig. 4:**
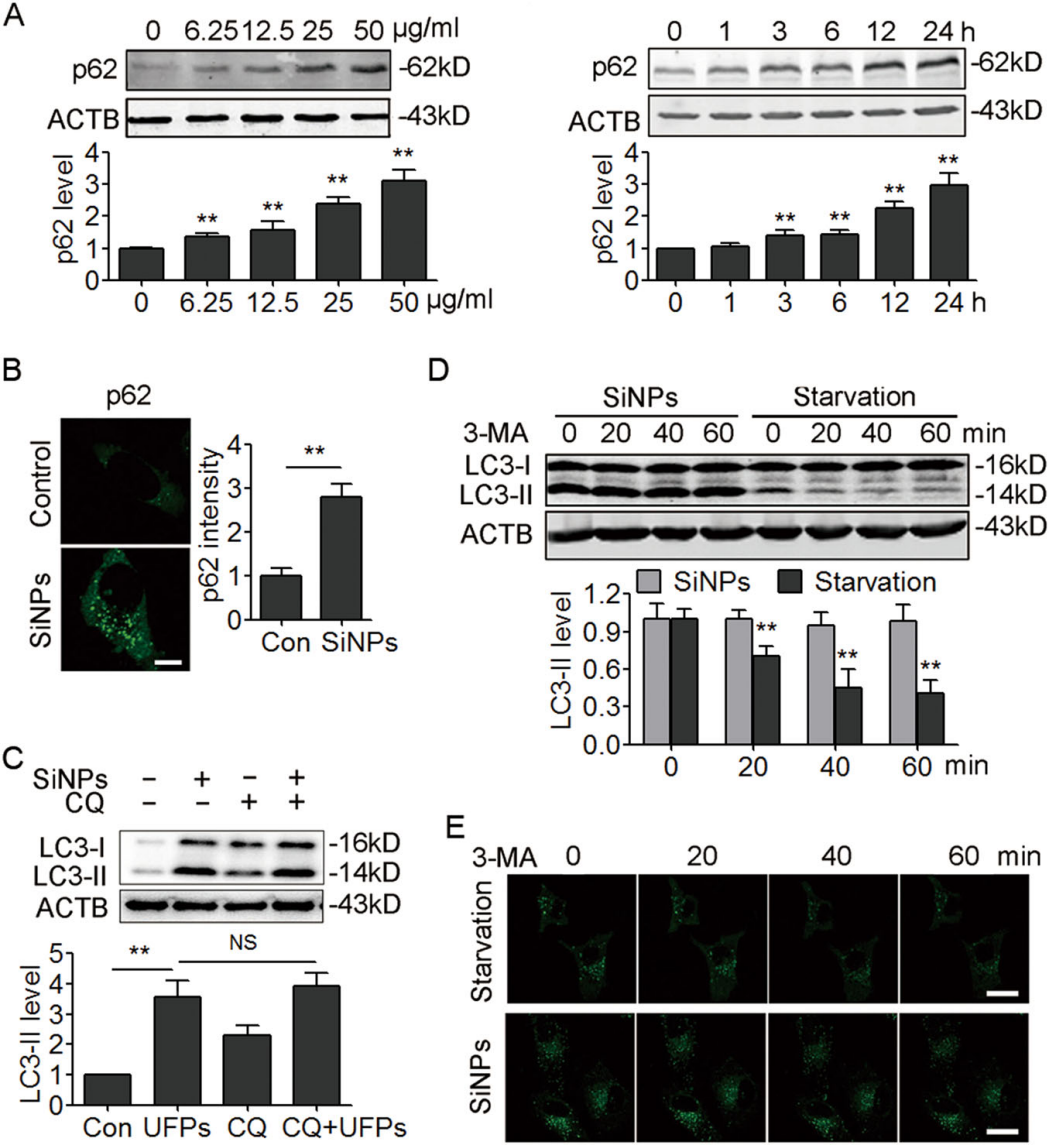
SiNPs inhibit autophagic degradation. **a** A549 cells were treated with different concentration of SiNPs as indicated for 24 h or with 50 μg/mL of SiNPs for different time as indicated. The cellular p62 expression was detected by western blot. The bottom panel shows the statistics for the relative p62 protein level. **b** A549 cells were treated with SiNPs at 50 μg/mL for 24 h. The expression of p62 was evaluated by indirect immunofluorescence assay. Quantification shown on the right represents the relative fluorescence intensity of p62. Scale bar: 5 μm, *n* = 50. **c** A549 cells were treated with SiNPs at 50 μg/mL in the absence or presence of CQ (20 μM) for 24 h, and the LC3 protein level was detected by western blot. The bottom panel shows the statistics for the relative LC3-II protein level. **d** A549 cells were treated with SiNPs at 50 μg/mL for 24 h or starvation medium, and then treated with 10 mM of 3-MA. The LC3 protein level was assessed by western blot at the indicated time. The bottom panel shows the statistics for the relative LC3-II protein level. **e** A549 cells transiently expressing GFP-LC3 were treated with 3-MA as in (**d**), and imaged by confocal microscopy. Scale bars: 10 μm. All quantitative data are presented as mean ± SEM, **P < 0.01

To further confirm that SiNPs trigger autophagy, the protein sequestosome 1 (p62), a substrate that is preferentially degraded by autophagy, was detected. Unexpectedly, the results revealed that SiNPs led to increase rather than decrease of p62 protein level in a dose- and time-dependent way (Fig. 4a, S2B), and that p62 proteins were assembled into aggregates in SiNPs-treated cells (Fig. 4b), suggesting that autophagic degradation is inhibited upon SiNPs treatment. Therefore, we performed an autophagic flux assay in the presence or absence of a lysosome degradation inhibitor, chloroquine (CQ). The results showed that CQ treatment did not further increase the LC3-II levels in SiNPs-treated cells (Fig. 4c, S2C), implying the blockage of autophagic flux by SiNPs.

We next directly evaluated the degradation of autophagosomes by applying 3-methyladenine (3-MA) to inhibit the synthesis of new autophagosomes, and detected LC3-II protein levels. Under 3-MA treatment, LC3-II was dramatically degraded in starved cells. However, LC3-II degradation was inhibited in SiNPs-treated cells (Fig. 4d). Autophagosome degradation was also tracked by time-lapse confocal microscopy in GFP-LC3 expressing cells. Similarly, we found that GFP-LC3 puncta were retained in SiNPs-treated cells after 3-MA treatment, further confirming an inhibition of autophagosome degradation (Fig. 4e). Thus, our data demonstrate that SiNPs block autophagic flux and autophagic degradation.

### SiNPs impair lysosomal degradation through inhibiting lysosomal acidification

**Fig. 5:**
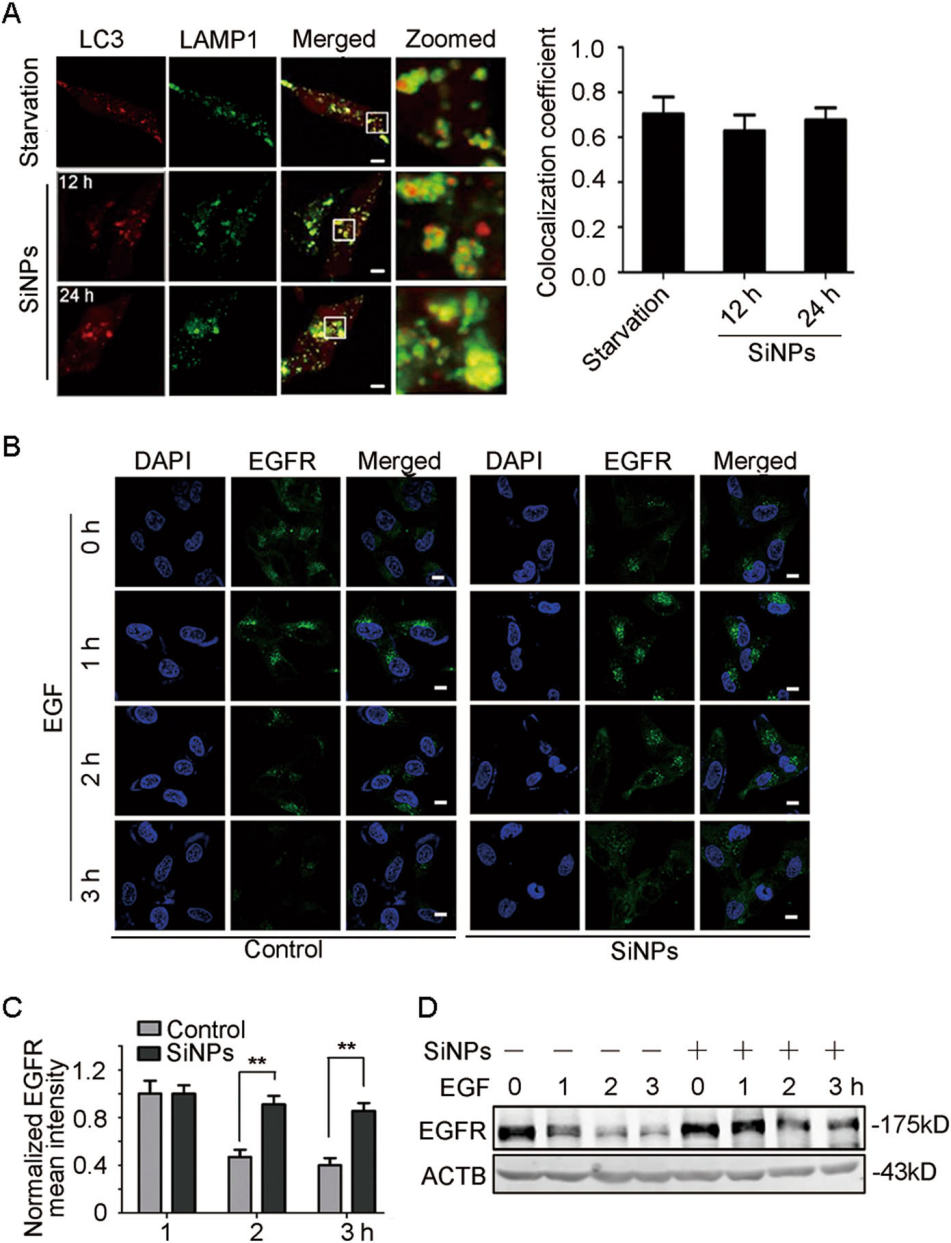
SiNPs impair lysosomal degradative capacity. **a** A549 cells transiently expressing CFP-LC3 and YFP-LAMP1 were either starved or treated with SiNPs at 50 μg/mL for 12 h or 24 h. The cells were then fixed and imaged by confocal microscopy. The graph on the right shows the statistics of the colocalization coefficients of LC3-CFP and LAMP1-YFP. Scale bars: 5 μm. **b** A549 cells treated with 50 μg/mL of SiNPs for 24 h were pre-incubated with 100 ng/mL of EGF for 15 min, and then the EGF was removed. At the indicated time after EGF treatment, the cells were fixed and immunostained with anti-EGFR antibody. Scale bars: 10 μm. **c** Quantitative analysis of panel B was performed by normalization to the intensity of cells with EGF treatment for 1 h (*n* = 30). **d** The EGFR protein level from the cells treated as in (**b**) was detected by western blot. Data are presented as mean ± SEM, **P < 0.01

After autophagosome formation, the complete autophagic process comprises their fusion with lysosomes and subsequent cargo degradation. We, therefore, first evaluated autophagosome-lysosome fusion by observing the colocalization of cyan fluorescent protein (CFP) tagged LC3 (CFP-LC3) and yellow fluorescent protein (YFP) tagged LAMP1. The results showed that the colocalization of LC3 and LAMP1 in SiNPs-treated cells was almost equal to starved cells (Fig. 5a), suggesting that SiNPs do not affect autophagosome-lysosome fusion. We then evaluated lysosomal degradation using an epidermal growth factor receptor (EGFR) degradation assay. The result revealed that EGFR puncta were largely retained in SiNPs-treated cells while a significant portion of the internalized EGFR was degraded in the control group (Fig. 5b, c). The immunoblotting assay also showed that SiNPs retarded cellular EGFR degradation (Fig. 5d), confirming a dysfunction of lysosomal degradation. These data indicate that SiNPs impair lysosomal degradation capacity.

**Fig. 6:**
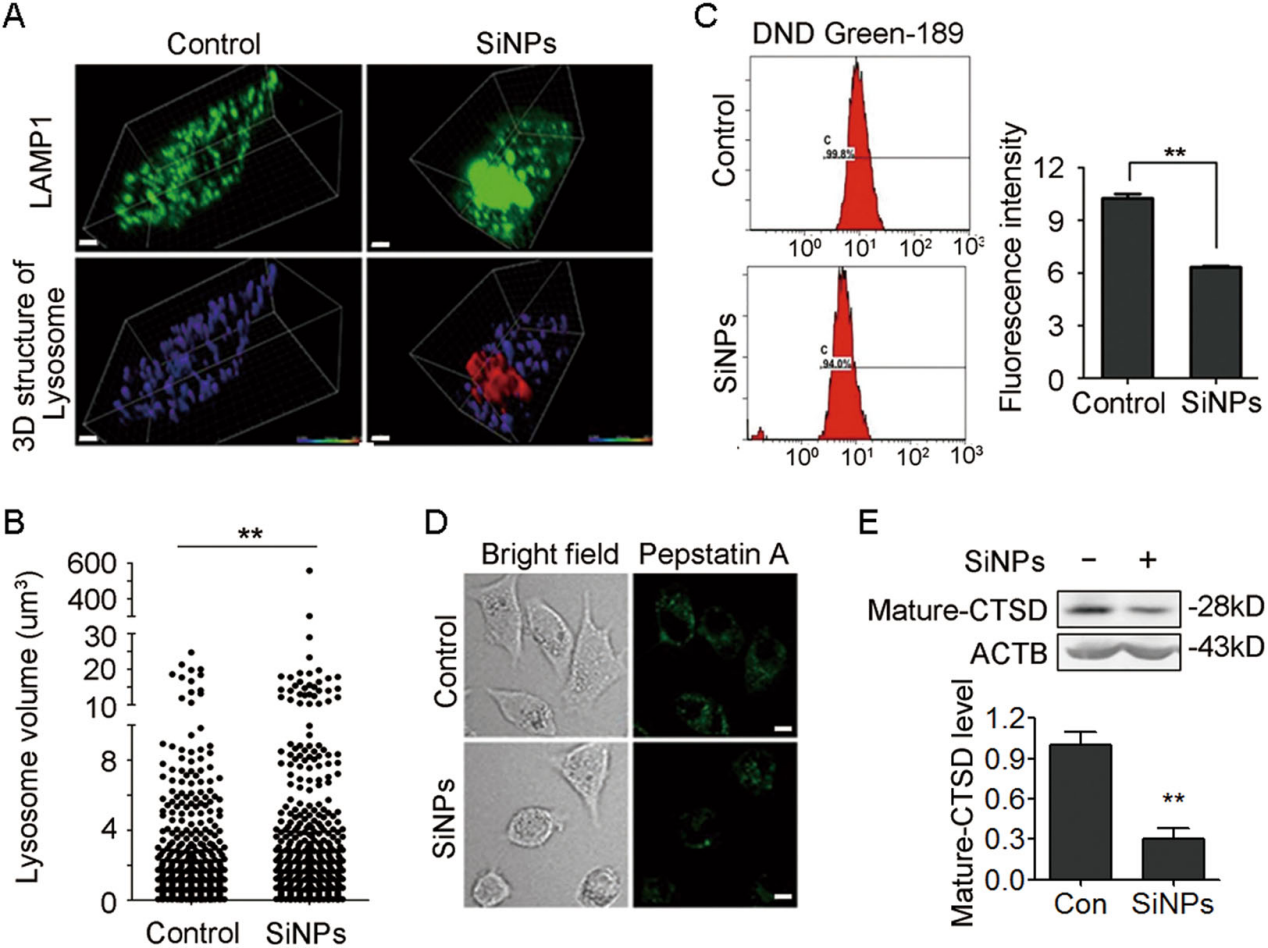
SiNPs enlarge lysosomes and inhibit lysosomal acidification. **a** A549 cells transiently expressing YFP-LAMP1 were treated with SiNPs at 50 μg/mL for 24 h. The cells were then fixed and imaged by confocal microscopy. The 3D structure of the lysosome were restructured with Imaris software. Scale bars: 4 μm. **b** The lysosome volumes of the cells treated as in (**a**) were quantified, and the difference between control and SiNPs treatment groups was tested with a Wilcoxon rank sum test. **c** A549 cells were treated with SiNPs at 50 μg/mL for 24 h, then stained with Lysosensor Green DND-189. The lysosensor fluorescence intensity was detected by flow cytometry. A representative flow cytometry profile is shown on the left. Quantification shown on the right represents the fluorescence intensity of the lysosensor probe. **d** A549 cells treated with SiNPs at 50 μg/mL for 24 h were incubated with 1.0 mM Bodipy-FL-pepstatin A for 1 h, and then visualized by confocal microscopy. Scale bars: 5 μm. **e** The mature CTSD protein level was assessed by western blot after SiNPs treatment as in (**d**). The bottom panel shows the statistics for relative mature-CTSD protein level. Data are presented as mean ± SEM, **P < 0.01

We further investigated the mechanism involved in lysosome impairment. The results revealed that the lysosome size was significantly increased (Fig. 6a, b), indicating a lysosome swelling. We next examined lysosomal acidification, a critical factor for lysosome maturation and activation of most lysosomal enzymes. Fluorescent Green-DND 189, a pH-dependent lysosensor, was introduced to the cells, and the fluorescence intensity was evaluated by flow cytometry. SiNPs treatment decreased the fluorescence intensity of Green-DND 189 (Fig. 6c, S2D), suggesting an increased lysosome pH. We further investigated the maturation of lysosomal enzymes under inhibition of acidification. Lysosomal hydrolase cathepsin D (CTSD) is a representative lysosomal enzyme, and Bodipy-FL-pepstatin A, a fluorescence dye, could selectively bind to mature active CTSD^30^. We found that mature CTSD levels were significantly decreased in SiNPs-treated cells (Fig. 6d). Immunoblotting analysis also demonstrated that SiNPs treatment resulted in a decline of mature CTSD protein level (Fig. 6e). These data indicate that SiNPs impair lysosomal acidification.

### Increasing autophagic degradation protects cells from SiNPs-triggered apoptosis

**Fig. 7:**
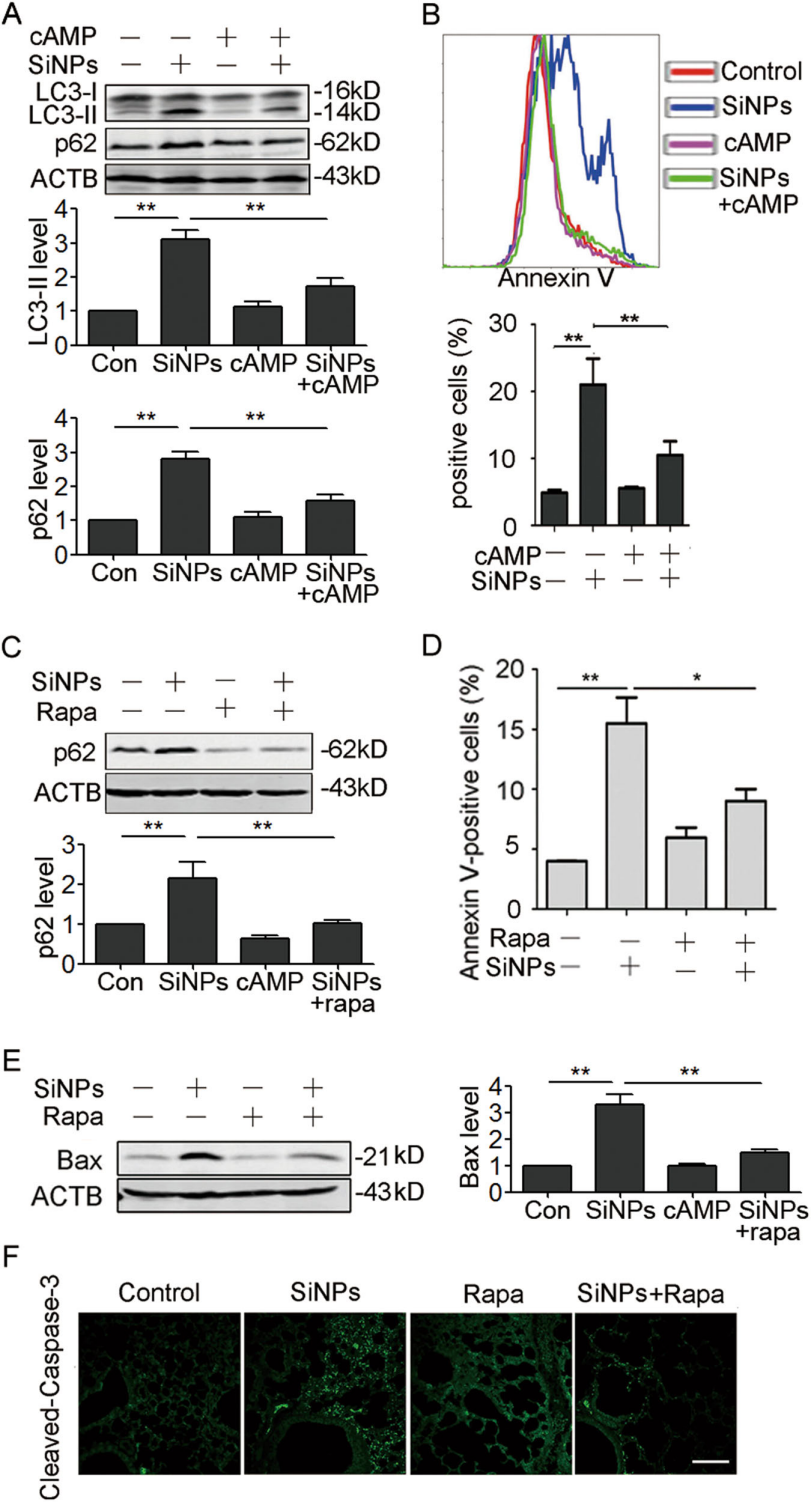
Increase of autophagic degradation protects cells from apoptosis. **a** A549 cells were exposed to SiNPs at 50 μg/mL, cAMP cocktail, or both. The expression of corresponding proteins was detected by western blot. The bottom panels show the statistics for relative LC3-II and p62 protein level. **b** A549 cells were treated with 50 μg/mL of SiNPs for 24 h in the presence or absence of cAMP cocktail. Cell apoptosis was monitored by flow cytometry analysis with Annexin V-fluorescein isothiocyanate (FITC) (top). The Annexin V positive cells were quantified (bottom). **c**, **d** A549 cells were treated with SiNPs in the presence or absence of rapamycin. **c** The p62 protein level was detected by western blot, and the bottom panel shows the statistical analysis for relative p62 protein level. **d** Cell apoptosis was monitored by Annexin V-FITC with flow cytometry analysis, and the Annexin V positive cells were quantified. **e**, **f** The mice instilled with SiNPs in the presence or absence of rapamycin for one month were harvested. **e** Bax expression in lung tissue was detected by western blot. The right panel shows the statistics for relative Bax protein level. **f** The Cleaved-Caspase-3 expression in lung tissue were detected by IF. Data are presented as mean ± SEM, *P < 0.05, **P < 0.01. Scale bars: 100 μm

To test whether the inhibition of autophagic degradation accounts for SiNPs-induced apoptosis, we performed rescue experiment. We used intracellular signaling molecule cyclic-3′,5′-adenosine monophosphate (cAMP) to restore lysosomal pH, and then evaluated lysosomal acidification, autophagic flux, and apoptosis^31^. Addition of cAMP indeed reduced lysosomal pH, and enhanced maturation of CTSD in SiNPs-treated cells (Figure S3A,B, Supporting Information). Furthermore, p62 and LC3-II levels in SiNPs-treated cells were significantly reduced in the presence of cAMP (Fig. 7a), suggesting that the reacidification of lysosomes enhances autophagic flux and promotes autophagic degradation. After cAMP treatment, cell viability loss (Figure S3C, Supporting Information), as well as cell apoptosis in SiNPs-treated cells were significantly attenuated (Fig. 7b).

Finally, we tested if a decreased autophagic degradation could directly account for apoptosis by enhancement of degradation with rapamycin. The results showed that rapamycin reduced p62 protein levels in A549 cells and significantly inhibited SiNPs-induced apoptosis (Fig. 7c, d). In addition, rapamycin also attenuated SiNPs-induced apoptosis in vivo (Fig. 7e, f). Collectively, our data indicate that SiNPs-induced apoptosis in AECs is a result of impared autophagic degradation.

### Decreased autophagic degradation participates in SiNPs-induced PF

**Fig. 8:**
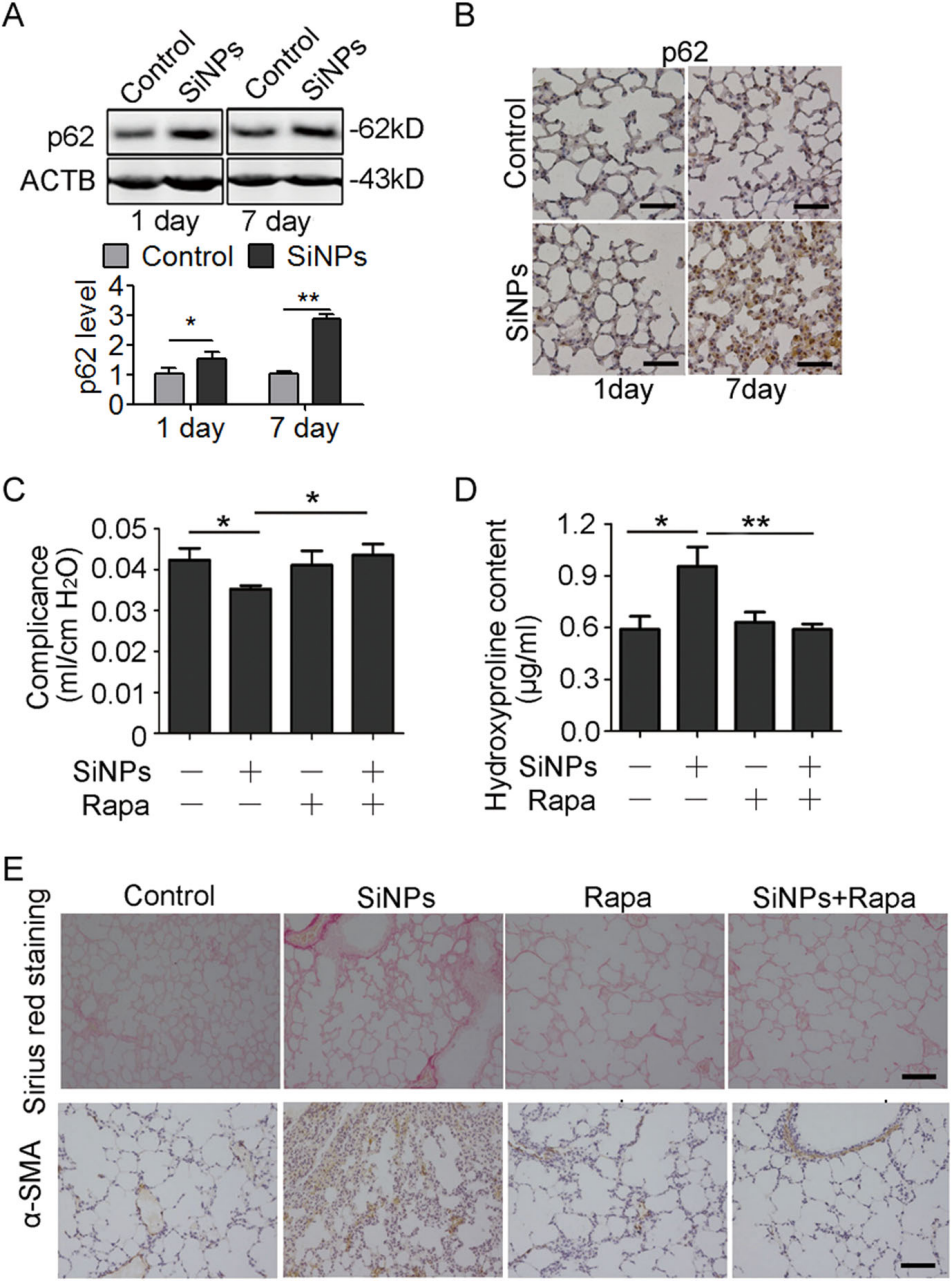
A decrease in autophagic degradation participates in SiNPs-induced PF. **a**, **b** The mice were instilled with PBS or SiNPs and harvested at the indicated time points. **a** p62 protein expression in lung tissue was detected by western blot. The bottom panel shows the statistics for the relative p62 protein level. **b** The expression of p62 was detected by IHC at 1 day or 7 days after instillation. **c**–**e** Mice were instilled with SiNPs in the presence or absence of rapamycin and harvested 1 month later. **c** Lung compliance was determined by Buxco system. **d** The HYP content in lung tissue was assessed by spectrophotometry. **e** The lung section was stained by Sirius red staining or performed IHC to detect α-SMA. Data are presented as mean ± SEM (n = 4 for each group), *P < 0.05, **P < 0.01. Scale bars: 100 μm

The observation that inhibition of autophagic degradation in A549 cells contributed to apoptosis implies a new mechanism underlying the pathogenesis of SiNPs-induced PF. To verify our in vitro findings, we examined the key molecule and core process in SiNPs-treated mice. Both immunoblotting and IHC assays showed increased p62 expression levels in the lung tissues (Fig. 8a, b), confirming that SiNPs inhibit autophagic degradation in vivo. We next interrogated whether enhancing autophagic flux in vivo could attenuate SiNPs-induced PF. For this purpose, intratracheally instilled mice were treated with rapamycin to activate autophagy and enhance autophagic flux^32^. Rapamycin improved pulmonary compliance while reduced HYP content, collagen deposition, as well as α-SMA levels in mice treated with SiNPs (Fig. 8c, d, e). These data demonstrate that enhancement of autophagic degradation could attenuate the SiNPs-induced PF, and further testify inhibition of autophagic degradation is involved in the pathogenesis of SiNPs-induced PF.

## Discussion

**Fig. 9:**
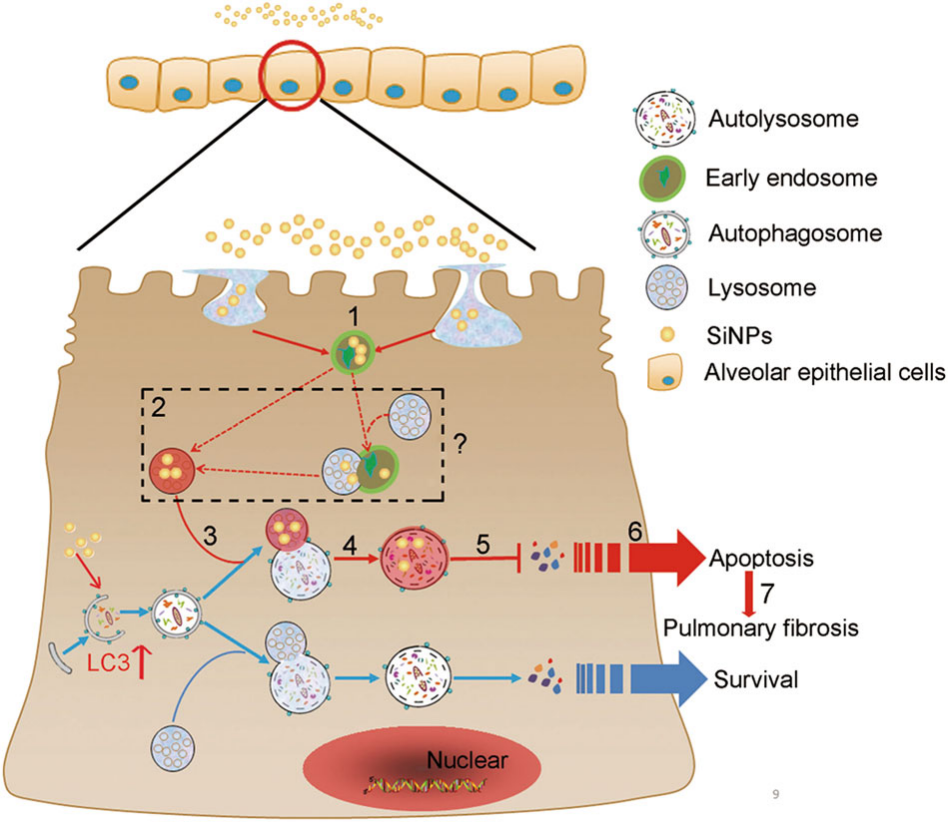
Schematic model for the mechanism of SiNPs-induced autophagy dysfunction in AECs and its role in the progression of SiNPs-associated PF. (1) SiNPs are endocytosed and located in early endosomes. (2) SiNPs enter lysosomes with unknown mechanisms (maturation of endosome to lysosome; or fusion of endosome with lysosome) and inhibit lysosomal acidification. (3) SiNPs do not affect autophagosome-lysosome fusion. (4) A dysfunctional autolysosome appears after autophagosome-lysosome fusion. (5) The autophagic degradation is blocked. (6) The autophagic flux blockage in AECs triggers apoptosis. (7) The apoptosis of AECs initiates PF

Although NPs have been reported to trigger PF development, its cellular and molecular mechanisms remain largely unclear. Previous studies mainly focus on the role of inflammation, ROS or cytokines in this process, but ignore the contribution of AEC autophagy and damage. In this study, we systematically investigated the mechanism of autophagy dysfunction in AECs and its role in SiNPs-induced PF. The major findings of our study include: (a) SiNPs entered AECs and resulted in autophagy dysfunction and apoptosis; (b) Autophagosome accumulation resulted from not only autophagy induction but also autophagic flux inhibition. Furthermore, SiNPs suppressed autophagic degradation by inhibiting lysosomal acidification and thus triggered apoptosis in AECs; (c) the reacidification of lysosome by cAMP cocktail or rapamycin enhanced autophagic flux, and protected AECs from apoptosis under SiNPs treatment; and (d) in vivo enhancement of autophagic flux by rapamycin attenuated SiNPs-induced apoptosis and PF. These findings demonstrate that impared autophagic degradation of AECs by SiNPs is involved in the progression of PF. Overall, our study elucidates a new mechanism in SiNPs-induced PF, namely that SiNPs inhibit autophagic degradation of AECs by impairing lysosomal acidification, thereby lead to apoptosis of AECs, and initiate PF, which can be rescued by enhancement of autophagic degradation (Fig. 9).

It is generally believed that NPs can target various cell types in lung tissue due to its nano-scale diameter, suggesting there are multiple target cells and mechanisms of action in NPs-induced PF. As a matter of fact, AEC injury and pneumocyte apoptosis have been reported to play roles in the development of PF, and the extent of AEC injury and lack of sufficient repair are critical determinants of PF following exposure to a wide variety of noxious agents^33–35^. However, the involvement of AEC apoptosis in NPs-induced PF has not been investigated. In this study, we not only provided evidence to support the alveolar type II cells as a target cell of SiNPs, but also elucidate a unique mechanism of SiNPs-induced cell apoptosis and subsequent PF. Based on our finding, we may conclude that SiNPs lead to AECs apoptosis through autophagy dysfunction, thus adding new knowledge to SiNPs-induced PF.

Autophagy have been implicated in a wide range of pulmonary disease, including PF^36^. In in vitro models, Araya et al. found that autophagy inhibition resulted in AEC senescence and myfibroblast differentiation, suggesting that insufficient autophagy is an underlying mechanism of both accelerated cellular senescence and myofibroblast differentiation in a cell-type-specific manner^37^. Using *Autophagy-related 4b* (*Atg4b*)-deficient mouse model, Cabrera et al. provided the first in vivo evidence that impaired autophagy augmented apoptosis predominantly in alveolar and bronchiolar epithelial cells, indicating that the ATG4B protease and autophagy play a crucial role in protecting epithelial cells against bleomycin-induced stress and apoptosis^18^. However, whether and how dysfunction of autophagy contributes to NPs-induced PF is still undefined. In this study, we demonstrated that accumulation of LC3-II protein in SiNPs-treated AECs were resulted from inhibition of autophagic flux and dysfunction of autolysosome degradation, indicating impairment of autophagy accounts for the AEC apoptosis and PF. Furthermore, we not only attested that SiNPs blocked autophagic degradation, but also elucidated that the underlying mechanism is lysosomal acidification inhibition. Wang et al. reported SiNPs-induced autophagy dysfunction via lysosomal impairment in hepatocytes, suggesting that autophagic degradation inhibition might be a common mechanism in various types of cells exposed to SiNPs^38^. Thus, we hypothesize SiNPs also damage other cells types (e.g., macrophages, fibroblasts) via autophagy dysfunction and contributed to SiNPs-induced PF. The other NPs, such as zinc oxide, gold nanoparticles as well as single-walled carbon nanotubes also damaged lysosome, therefore inhibited autophagic flux to lead to cytotoxicity, indicating that autophagy dysfunction resulting from lysososmal impairment acts as a general toxic mechanism of NPs^39–41^. It also should be noted that SiNPs upregulated *LC3* mRNA level, leading to more LC3 than CQ treatment, suggesting that SiNPs not only impared autophagic degradation, but also played a role in autophagy induction (Figure S4).

Interestingly, our finding is similar to the so-called lysosomal storage disorders (LSDs), which are a heterogeneous group of inherited diseases, resulting from deficiencies of one or more enzymes or transporters that normally reside within the lysosomes^42^. In several LSDs, the accumulation of autophagosomes has been contributed to a decreased autophagic flux^43,44^. Furthermore, perturbations of autophagic flux by specific gene mutations have been suggested to contribute to the disease etiology of specific LSDs. Based on lysosomal dysfunction and decreased autophagic degradation, SiNPs-induced PF might be defined as a type of LSD-like disease^45^. Collectively, the LSD-like diseases are caused by various stimuli that impair the lysosome, inhibit autophagic flux and thus trigger subsequent injury.

NPs exposure could lead to PF, therefore, it is important to develop intervention measures for prevention and treatment of NPs-stimulated PF. Currently, the treatment of PF continues to pose major difficulties. Until recently, most treatment options have focused on inflammation by using anti-inflammatory agents^46^. However, the strategy has been found to be effective only in small groups of patients^47^. More new targets, for example, PF-associated cytokines, have been deciphered along with more understanding of the underlying pathophysiology of PF. However, because of multiple routes for PF stimulation, blocking one of them had no significant effect on the ultimate outcome^48^. Promoting the prevention of epithelial damage/healing of the lung epithelium has been increasingly investigated as a potential approach by which subsequent inflammation, remodeling and fibrosis can be abrogated. Therefore, there is a need to develop therapies that promote the protection and health of lung epithelial cells. In the present study, we paid our attention to AEC damage under SiNPs treatment, and clarified the underlying mechanism of autophagy dysfunction in AEC damage, thus enriched our understanding of the pathogenesis of this disease. Therefore, our results provide not only a new strategy targeting autophagy flux blockage to prevent SiNPs-induced AEC damage and PF, but also experimental data for a preventive effect of autophagic degradation enhancement with rapamycin in SiNPs-treated mice.

In summary, for the first time, we have shown that autophagy participates in SiNPs-induced PF. We have also identified that the autophagic flux blockage results from lysosomal acidification inhibition, which then triggers apoptosis in AECs and subsequent PF. These findings provide a new mechanism by which SiNPs trigger PF by targeting AECs. Furthermore, these results may lead to new strategies to prevent SiNPs-induced PF by enhancing autophagic degradation.

## Supplementary information

Supplementary Material
