# Supplementary material for: Autophagic flux blockage in alveolar epithelial cells is essential in silica nanoparticle-induced pulmonary fibrosis
Source: Cell Death Dis. 2019 Feb 12;10(2):127. doi: 10.1038/s41419-019-1340-8 (PMC6372720; doi:10.1038/s41419-019-1340-8)
Supplement: Supplementary file 1 — Supplementary Material [file 41419_2019_1340_MOESM1_ESM.docx]

**Supplementary Materials**

Autophagic Flux Blockage in Alveolar Epithelial Cells is Essential in Silica Nanoparticle-Induced Pulmonary Fibrosis

Xinyuan Zhao^1,5,#^, Saisai Wei^1,#^, Zhijian Li^3^, Chen Lin^1^, Zhenfeng Zhu^4^, Desen Sun^1^, Rongpan Bai^1^, Jun Qian^4^, Xiangwei Gao^1*^, Guangdi Chen^1*^, Zhengping Xu^1,2*^

1 Institute of Environmental Medicine, Zhejiang University School of Medicine, Hangzhou 310058, China;

2 Collaborative Innovation Center for Diagnosis and Treatment of Infectious Diseases, Zhejiang University, Hangzhou 310058, China;

3 The First Affiliated Hospital, Zhejiang University School of Medicine, Hangzhou 310058, China;

4 State Key Laboratory of Modern Optical Instrumentation, Centre for Optical and Electromagnetic Research, JORCEP (Sino-Swedish Joint Research Center of Photonics), Zhejiang University, Hangzhou 310058, China;

5 Department of Occupational Medicine and Environmental Toxicology, School of Public Health, Nantong Unversity, Nantong 226019, China.

# These authors contributed equally to the work.

* Correspondence to: Zhengping Xu, Email: zpxu@zju.edu.cn; Guangdi Chen, Email: chenguangdi@zju.edu.cn; or Xiangwei Gao, Email: xiangweigao@zju.edu.cn

**Supplementary Figures**


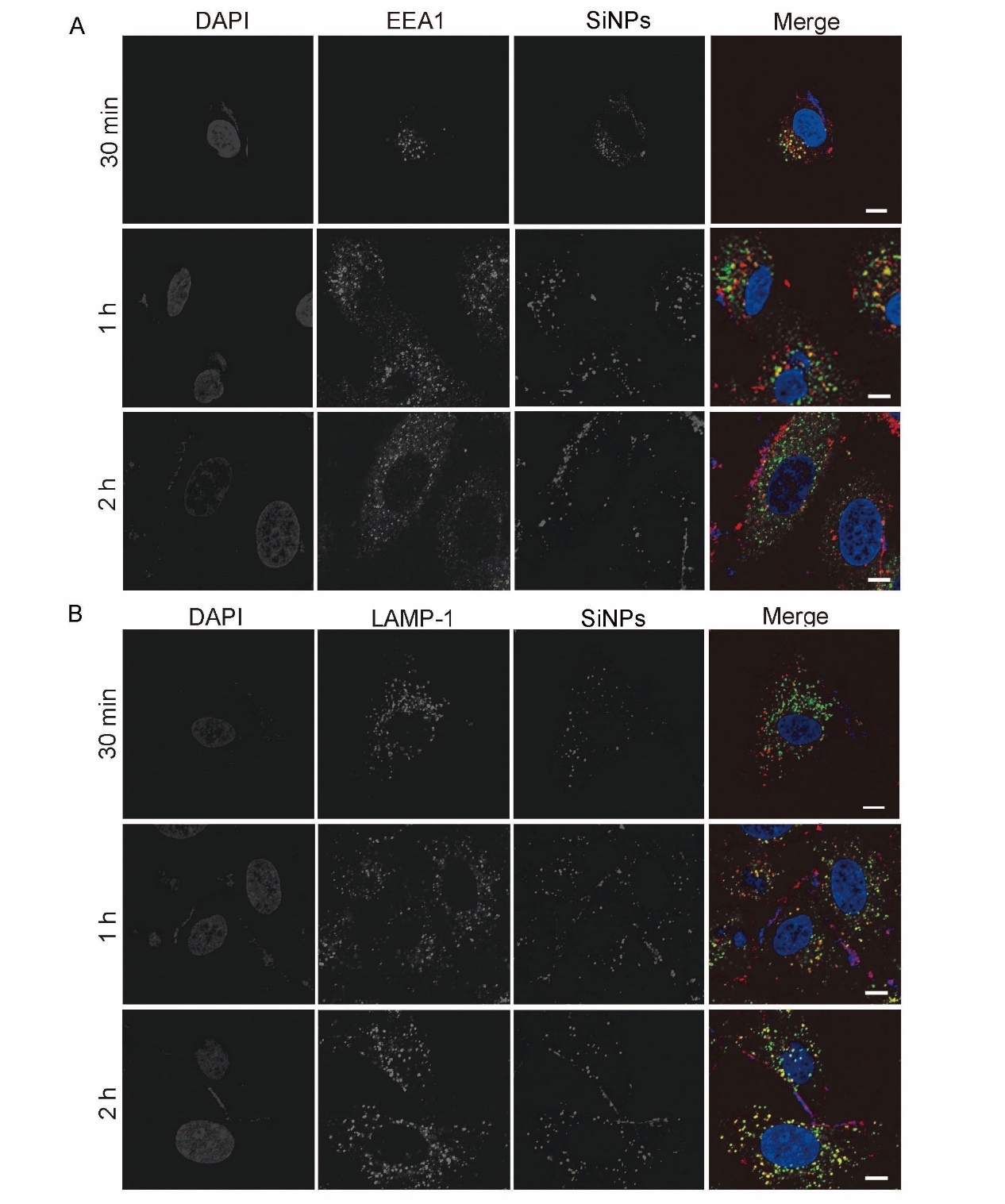


**Figure S1.** SiNPs first locate in early endosomes then exist in lysosomes. (A) A549 cells treated with SiNPs (red) for indicated time then stained with EEA1 (green). (B) A549 cells treated with SiNPs (red) for indicated time then stained with LAMP-1(green). Scale bar: 5 μm.


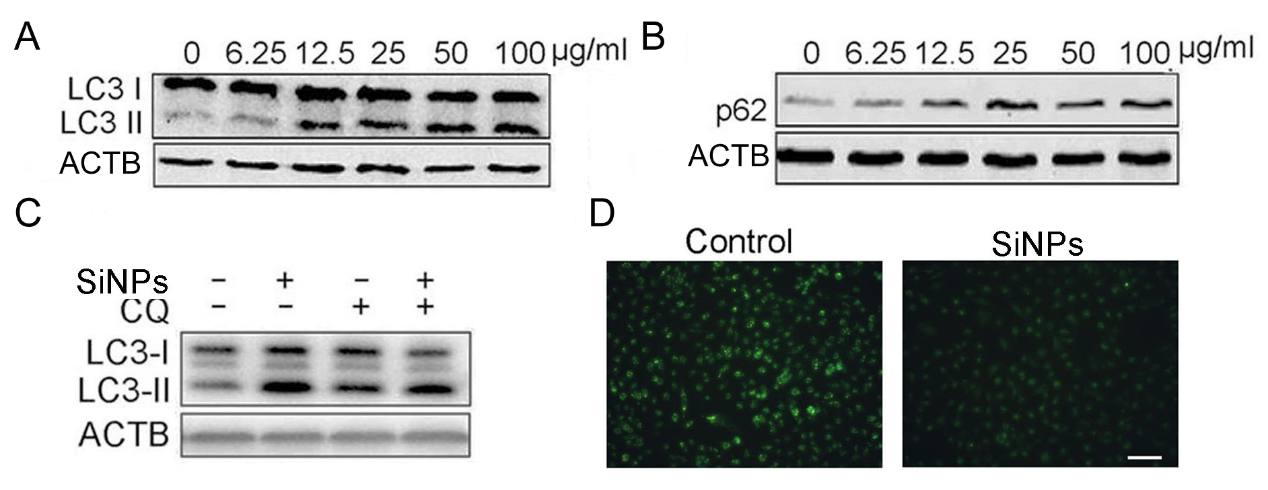


**Figure S2.** The effects of SiNPs on autophagic degradation and lysosomal pH in BEAS-2B cells. (A,B) BEAS-2B cells were treated with different concentrations of SiNPs for 24 h. The cellular LC3 (A) and p62 (B) protein expression was detected by Western blot. (C) BEAS-2B cells were treated with SiNPs at 50 μg/mL in the absence or presence of CQ (20 μM) for 24 h, and the LC3 protein levels were detected by Western blot. (D) The lysosomal pH level of BEAS-2B cells with or without SiNPs treatment was detected by imaging Lysosensor Green DND-189 probe. Scale bars: 200 μm.


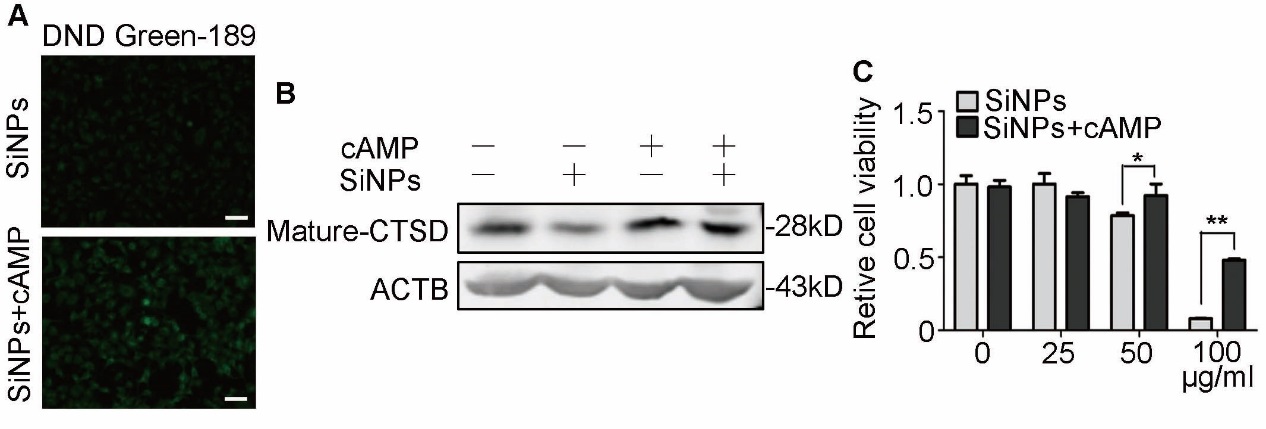


**Figure S3.** cAMP treatment restores lysosomal pH, enhances maturation of CTSD, and protects cells from viability loss. A549 cells were treated with SiNPs and then followed by cAMP treatment. (A) The lysosomal pH level was detected by imaging Lysosensor Green DND-189 probe. Scale bars: 50 μm. (B) Mature-CTSD levels were accessed by Western blot. (C) The cell viability was detected by CCK-8.


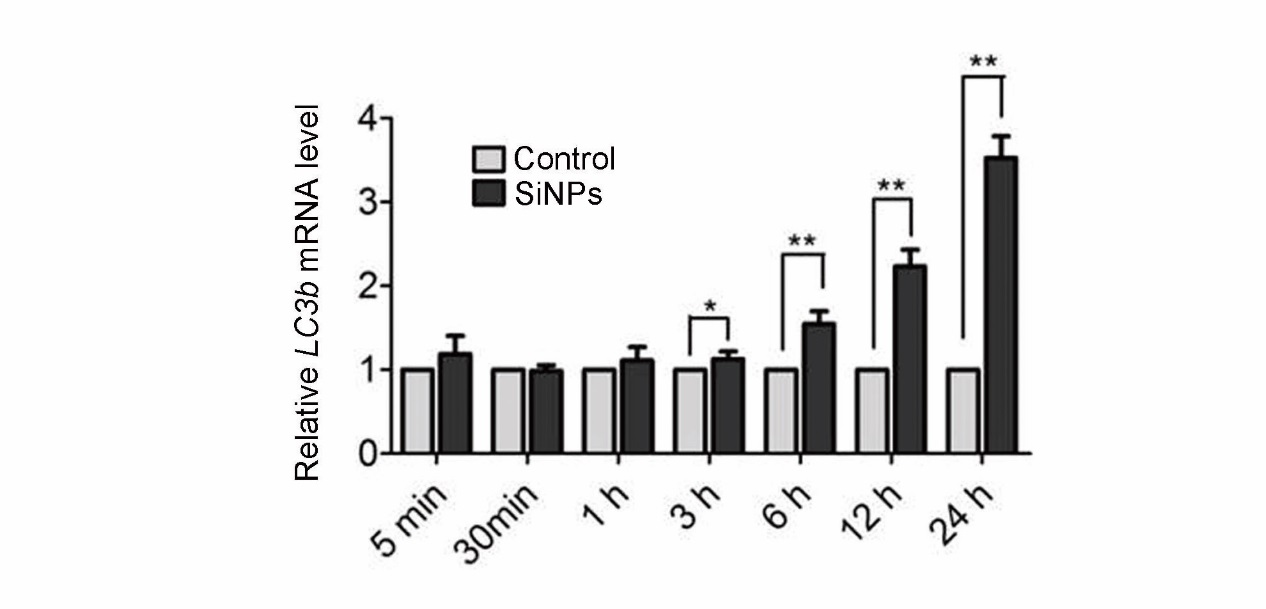


**Figure S4.** SiNPs increase LC3 expression. A549 cells were treated with SiNPs at 50 μg/mL for different durations as indicated, and *LC3* mRNA expression levels were assessed by qRT-PCR.
